# Green space and older adults’ health: a scoping review

**DOI:** 10.1093/geroni/igaf119

**Published:** 2025-10-30

**Authors:** Qi Wang, Jingxing Song, Zhaoqi Liu, Cheng Shi

**Affiliations:** Centre on Behavioral Health, The University of Hong Kong, Hong Kong, China; School of Graduate Studies, Lingnan University, Hong Kong, China; Landscape Architecture Department, School of Architecture and Urban Planning, Shenzhen University, Guandong, China; School of Graduate Studies, Lingnan University, Hong Kong, China; Institute of Policy Studies, Lingnan University, Hong Kong, China

**Keywords:** Greenspace, Blue space, Mental wellbeing, Urban planning

## Abstract

**Background and Objectives:**

Previous research has highlighted the connections between individuals’ exposure to green spaces and various health outcomes. However, the unique post-retirement context of older adults, characterized by changes in activity patterns and mobility, has not been thoroughly examined on a global scale. This scoping review aims to map the evidence on how different representations and metrics of green spaces relate to diverse health outcomes among older adults and to identify the key factors that differentiate these relationships.

**Research Design and Methods:**

Following Arksey and O’Malley 5-stage framework, a systematic search was conducted in 4 major databases: Web of Science, PubMed, Scopus, and EBSCO. Articles were included if they focused on green space exposures and older adults’ health outcomes.

**Results:**

A total of 40 studies, primarily cross-sectional and conducted in different countries, were included. These studies spanned a wide range of green space metrics and health outcomes. Overall, they consistently found that exposure to green spaces correlates with improved perceived physical and mental health among older adults, while these relationships vary due to the influence of confounders, moderators, and medicators.

**Discussion and Implications:**

The findings suggest a positive association between green space exposure and health among older adults, though the cross-sectional nature of most included studies limits the ability to establish causality. This review also outlines potential pathways linking green spaces to health benefits, providing a comprehensive framework for future investigations. These insights can guide further research, particularly longitudinal or intervention-based studies, to deepen our understanding of how green spaces can promote healthy aging.

**Innovation and Translational Significance:** This scoping review examines the relationship between green spaces and older adults’ health, addressing gaps in existing research. It highlights that exposure to green spaces is positively associated with improved physical, mental, and social health outcomes in older adults, facilitated by pathways such as physical activity, stress reduction, and social interaction. However, most studies are cross-sectional, limiting causal inferences. The review emphasizes the need for tailored interventions, considering accessibility, mobility, and social needs, to maximize benefits for older adults. Future research should explore mediators, moderators, and longitudinal impacts to better inform urban planning and public health strategies.

Green space has long been recognized as a vital determinant of public health (Zhang et al., 2022). In recent decades, the relationships between green spaces and health-related behaviors and outcomes have been thoroughly investigated by health professionals, urban planners, public health analysts, and policymakers, demonstrating positive correlations across numerous studies (Aggio et al., 2015; Akpinar & Cankurt, 2017; Helbich et al., 2018; Ji et al., 2019). Thus, understanding how green spaces improve health and well-being is crucial for creating healthy cities and promoting public health, especially in the context of rapid urbanization worldwide (United Nations, 2019).

A substantial number of primary studies and systematic reviews have evidenced a positive relationship between urban green elements and the health of nearby residents. Specifically, green space exposure is linked to improved physical health, including lower risks of obesity, diabetes, and cardiovascular disease (Astell-Burt et al., 2021), reduced heat-induced mortality (Madrigano et al., 2015), and better maternal and infant health (Xiao et al., 2021). Mental health benefits include reduced stress, depression (Roberts & Helbich, 2021), and anxiety (Hartley et al., 2021), with meta-analyses showing a 10% increase in green space correlating with lower depression risk (odds ratio [OR] = 0.963) (Liu et al., 2023). Green space also influences social determinants of health, such as safety perceptions (Kondo et al., 2021), food security (Guezo, 2014), and inequalities in access (Zhang et al., 2021). Systematic reviews confirm associations with lower cortisol, blood pressure, and all-cause mortality (Twohig-Bennett & Jones, 2018), reinforcing green space’s role in sustainable urban development (SDG-11) (Rebecchi & Capolongo, 2021).

One scoping review (Liu et al., 2023) has identified seven possible pathways through which green space, both as a complete entity and through its six components, can impact human’s physical, mental, and social health. First, green space as a complete entity can serve as a vital recreational area in urban environments, promoting physical activity (Chang et al., 2020; Yuen et al., 2019) and social interactions while enhancing mental well-being through aesthetic appeal (Pretty et al., 2005). Second, the components of green space include air quality, sound, blue space, wildlife, soil, and micro-climate (Liu et al., 2023). For example, vegetation mitigates urban heat islands, reducing heat-related mortality (Madrigano et al., 2015), while soil may contain pollutants like polycyclic aromatic hydrocarbons and heavy metals (Lin et al., 2018; Zhang et al., 2016). Green space also interacts with water resources—evapotranspiration cools urban areas but may exacerbate water scarcity (Yin et al., 2021), and irrigation with recycled wastewater can introduce contaminants (Lyu et al., 2016). Wildlife in green spaces poses risks of zoonotic diseases and conflicts (Blasdell et al., 2022; Hosaka & Numata, 2016), though bird sounds enhance mental restoration (Uebel et al., 2021). Green spaces filter air pollutants like PM2.5 (Kwon et al., 2021) but may produce allergenic pollen (Suanno et al., 2021). Microbial exposure may boost immune resilience (Selway et al., 2020) or increase infection risks.

Despite the growing body of research on the relationship between green space and health and the publication of the umbrella review of green space and human health (Yang et al., 2021), notable deficiencies persist in the current literature. A significant gap is that the majority of meta-analyses and systematic reviews have focused on the general population or adolescents and youth (Ye et al., 2022; Zhang et al., 2020), while neglecting the older adult demographic and failing to particularly address their needs. This cohort may derive greater advantages from exposure to natural surroundings owing to their distinct health and mobility requirements, perhaps more so than other demographic groups. As they transition into retirement, their daily routines often shift, and the exposure to green spaces provides an opportunity for physical activity, relaxation, and social interaction, all of which are vital for maintaining physical and mental health (Enssle & Kabisch, 2020; Hong et al., 2018; Petersen et al., 2018). The natural environment can stimulate the senses, boost mood, and reduce stress, thereby improving the overall quality of life. Additionally, green spaces promote mobility and independence, crucial aspects that can enhance an older adult’s sense of self-efficacy and well-being (Yu et al., 2024). The accessibility of these spaces can also mitigate feelings of isolation or loneliness that some older adults may experience (Wiles et al., 2009). Therefore, the benefits derived from green spaces are multifaceted and particularly crucial for the older population. As the global population ages rapidly, with the number of people aged 60 and older projected to increase significantly, it is essential to comprehend how green spaces might uniquely influence older folks and address their particular needs.

A few review studies examine the relationship between green space and older adults’ wellbeing; however, there is a lack of comprehensive scoping reviews that examine different measures of green space and various health outcomes among older adults. Current reviews (Chen et al., 2021; Levy-Storms et al., 2018; Yuan et al., 2021) only examined the associations between green space and physical health or green space between mental health but ignored health as a holistic concept for humans. A thorough investigation is needed to understand how different types and qualities of green spaces influence physical, mental, and social health outcomes in older adults. Such an examination would provide a more detailed understanding of the specific attributes of green spaces that are most beneficial for this demographic.

Also, moderating and mediating factors that link green space to various health outcomes have not been comprehensively synthesized among studies focusing on older adults. A scoping review by Dzhambov et al. (2020) identified the most frequently examined mediators between green space and health among the general population were physical activity and air pollution. For cardiometabolic outcomes, most studies explored mediation through these two factors; in studies on birth outcomes, air pollution was tested and consistently considered as a mediator, while for mortality and cognitive function, air pollution was also tested as a mediator in most studies. Additional variables receiving increasing attention include perceived green space quantity and the amount of contact with green space (Dahlkvist et al., 2016; Dzhambov et al., 2018; Yang et al., 2020), perceived restorative quality (e.g., Dahlkvist et al., 2016; Dzhambov et al., 2018), cognitive resources (Kuo, 2001; Panno et al., 2017; Van Herzele & de Vries, 2012), and connectedness to nature or environmental satisfaction (Bakir-Demir et al., 2019; Mukherjee et al., 2017; Preuß et al., 2019; Van Herzele & de Vries, 2012). In addition, traffic noise has also been considered as a potential mediator. Although some studies have observed an indirect effect of traffic noise on mental health (Dzhambov et al., 2018), the overall evidence does not strongly support its role as a mediator (Cusack et al., 2018; Dzhambov et al., 2018; Hystad et al., 2014; Markevych et al., 2014). Moreover, some scholars also tested several mediators in one study. For example, Ulmer et al. (2016) examined seven potential mediators of the relationship between green space and general health, including distress, social cohesion, physical activity, obesity, hypertension, diabetes, and asthma. Similarly, Klompmaker et al. (2019) independently assessed several variables previously identified as causally related, such as air pollution, traffic noise, and various chronic diseases. The current review lacks comprehensive analyses of both moderating and mediating factors, which could provide deeper insights into the mechanisms through which green spaces impact health for older adults. Understanding these mechanisms is essential for tailoring interventions to maximize the benefits of green spaces for older adults. This gap highlights the need for research that moves beyond simple associations to explore the underlying processes connecting green space exposure to health outcomes, particularly for vulnerable populations like older adults. Such investigations would help develop more targeted and effective public health strategies.

Moreover, buffer zones, which refer to the geographical areas around green spaces that might influence their accessibility and usage, play a critical role in measuring green space exposure. However, their optimal sizing remains unconclusive for older adults. Research suggests two effective ranges: smaller buffers (100–500 m) capture local, walkable green spaces, while larger buffers (2,000–5,000 m) reflect broader environmental contexts (Liu et al., 2025). These zone sizes significantly influence exposure measures and subsequent health impact analyses, with context-specific buffers needed to minimize both commission errors (including irrelevant green spaces) and omission errors (excluding relevant ones). The selection of appropriate buffer sizes is especially crucial for older populations, as their reduced mobility makes smaller, proximal buffers more meaningful for accessibility assessments. Current findings demonstrate spatial non-stationarity in green space–health relationships, rejecting a one-size-fits-all approach (Liu et al., 2025). A review study is needed to validate optimal buffer zones and their health impacts, ultimately informing more precise urban planning strategies that enhance green space accessibility for aging populations.

To address these gaps, we conducted a scoping review to map the breadth of existing evidence on the relationship between green space and older adults’ wellbeing and identify factors differentiating this relationship. Specially, we categorize available representations and metrics of green spaces that can be used to assess older adults’ exposure to such spaces and examine their relationship with diverse health outcomes. This scoping review aims to enhance our understanding of how green spaces can be leveraged to improve the health and well-being of older adults. This, in turn, we aim to provide valuable insights that inform the creation of healthier, more supportive environments for older adults and lead to more targeted and effective public health and urban planning interventions.

## Method

This review followed the Preferred Reporting Items for Systematic reviews and Meta-Analyses (PRISMA) guidelines. Reporting of the study flow and findings was in accordance with the PRISMA statement (Shamseer et al., 2015). A five-stage methodology was adopted (Arksey & O’Malley, 2005), in order to produce a comprehensive review of the included studies, including identifying the research question, identifying relevant studies, selecting studies, charting the data and collating, summarizing, and reporting results.

### Identifying the research question

Given the identified research gaps, this study aims to examine the categorization of green spaces, health outcomes, and the relationship between green spaces and health among older adults who may derive greater benefits from such spaces. Furthermore, exploring the pathways linking green spaces and health is also of significant importance. Thus, the guiding research question for this scoping review is, What is the relationship between green spaces and the health outcomes of older adults worldwide, and what potential moderators and mediators exist?

### Search strategy

During the search process, four major academic databases were utilized: Web of Science (WoS), PubMed, Scopus, and EBSCO. The search strategy was based on Boolean logic (OR and AND) and involved retrieving articles published in English from any year up to December 2023. Search terms included “Green space,” “Health,” and “Older people,” with their exchangeable terms detailed in Supplementary Table 1 (see online supplementary material). The search terms were applied across all databases and were tailored to fit the relevant keyword fields specific to each database.

### Study selection

#### Eligibility criteria

The inclusion criteria were (1) Population: People aged 60 and over (the older population must be clearly separated); (2) Study design: Quantitative study; review articles were included in the title and abstract screening but excluded in the full-text review (to ensure that potentially relevant primary studies referenced or mentioned within the reviews were not missed during the initial screening process); (3) Location: Any geographic location in both rural and urban areas; (4) Outcome: Outcomes related to physical/mental health, well-being, or quality of life; (5) Intervention: Green space; (6) Year: Any year; and (7) Publication language: English; (8) Publication type: peer-reviewed journal articles. The exclusion criteria were (1) Population: People aged below 60 years old; and (2) Study design: Theoretical articles; qualitative studies and review studies; (3) Publication type: non-peer reviewed articles (the focus of this study was solely on academic papers to ensure a consistent and rigorous analysis based on peer-reviewed work). Thesis, conference abstracts were excluded by their reference lists was checked.

#### Selection of studies

Study selection was conducted by two independent reviewers in two stages: initial title and abstract screening, followed by full-text review. Duplicate records were removed during import into Rayyan (https://rayyan.qcri.org/), a collaborative screening platform (Ouzzani et al., 2016). Prior to the selection process, the team, comprising four experienced researchers, thoroughly discussed the inclusion and exclusion criteria to ensure a consistent understanding. Conflicts arising from the title and abstract screening were resolved through discussion among the four reviewers (L.Z., S.C., W.Q., and S.J.). The full texts of the included studies were reviewed by two reviewers (S.Y. and S.J.), with any conflicts addressed by a third reviewer (S.C.) from the research team.

### Quality appraisal

Given that the purpose of this scoping review was to offer a broad and descriptive summary of the existing evidence (Munn et al., 2018), the article did not undergo quality assessment.

### Data charting

Data charting and organization of our scoping review were guided by a recent methodological scoping review (Tricco et al., 2016). Data on the included study characteristics were extracted: name of the first author and the publication year; sample characteristics (number of participants, share of men/women, setting, and country); study design; green space, health outcomes, mediating and moderating variables; and reporting of results (major findings). These data were extracted by one reviewer and verified by another reviewer.

## Results

### Overview

**Figure 1.**
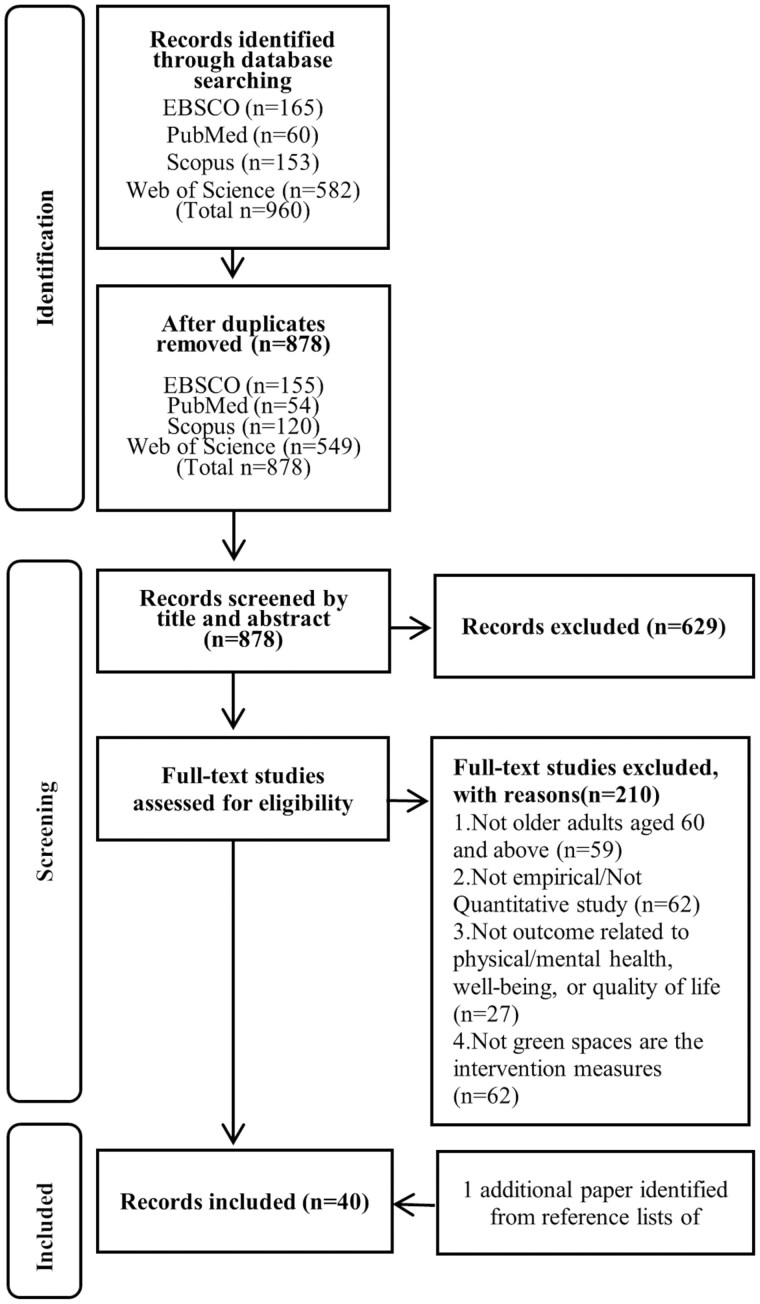
Flow chart of study selection.

During the database retrieval process, a total of 960 articles were retrieved. After removing duplicates, 878 articles were included in the initial screening. Following screening based on titles and abstracts, 249 articles were included for full-text review. After the full-text review, a final set of 39 articles were included for summary analysis. One more study was added after a grey literature search. Figure 1 illustrates the selection process of the 40 included articles.

This scoping review included 40 studies in which the relationship between green space and older adults’ health was explored. As shown in Supplementary Table 2 (see online supplementary material), details of these studies are listed. All selected studies were published between 2005 and 2024. The 40 included studies were conducted among older adults across various countries, including Iran (study IDs: 1, 17), the United States (study IDs: 2, 3, 26), Ireland (study ID: 4), Hong Kong (study IDs: 5, 18, 22, 36, 38), Mainland China (study IDs: 6, 7, 9, 12, 13, 14, 15, 16, 20, 21, 29, 34, 35, 39, 40), Sweden (study ID: 8), India (study ID: 10), the United Kingdom (study IDs: 11, 33), Korea (study ID: 19), Brazil (study ID: 23), Finland (study IDs: 24, 25), Japan (study ID: 27), Hong Kong and Taiwan (study ID: 28), the Netherlands (study ID: 30), Germany (study ID: 31), Hong Kong and Germany (study ID: 32), and Macau (study ID: 37).

The studies also recruited participants of different age groups, including those 65 years and above (study IDs: 1, 2, 3, 5, 9, 11, 19, 22, 27, 31, 36, 37, 38), 50 years and above (study IDs: 4, 8), 60 years and above (study IDs: 6, 7, 12, 13, 14, 15, 17, 18, 20, 21, 23, 25, 29, 32, 34, 39, 40), older adults without a specific age (study ID: 10), 80 years and above (study ID: 16), 70 years and above (study ID: 24), 75 years and older (study ID: 26), 55 years and above (study IDs: 28, 35), over 30 years with a mean age of 61.5 and 55.9 (study ID: 30), and 74 years and above (study ID: 33).

Various methodologies were employed in these studies, including first-hand cross-sectional field surveys (study IDs: 1, 5, 6, 7, 8, 13, 17, 18, 20, 24, 25, 28, 29, 30, 32, 33, 34, 38, 39, 40), second-hand cross-sectional data analysis (study IDs: 2, 3, 4, 11, 12, 14, 15, 19, 21, 23, 26, 27), experimental studies (study IDs: 9, 35), mixed-method studies (study ID: 10), first-hand longitudinal studies (study IDs: 22, 36), second-hand longitudinal data (study ID: 16), and multi-wave second-hand data analysis (study ID: 31).

The participants included community members (study IDs: 1, 6, 7, 8, 9, 10, 12, 13, 14, 15, 16, 17, 18, 19, 20, 21, 22, 23, 26, 28, 29, 31, 32, 33, 34, 36, 37, 38, 39, 40), those with annual clinic visits (study ID: 2), residents of state-certified nursing home care facilities (study ID: 3), the general population of men (study ID: 11), residents of nursing homes and service centers (study ID: 24), female residents of nursing homes and service centers (study ID: 25), older adults who had already been certified as needing long-term care (study ID: 27), and all-female participants (study ID: 35). Sample size of the included studies ranged from 30 (study ID: 24) to 368,399 (study ID: 14).

In the studies included and reported sex ratio of participants, there were studies included more men than women (study IDs: 1, 17, 18, 26, 29, 36), an equal number of men and women (study IDs: 9, 22), more women than men (study IDs: 2, 3, 4, 5, 8, 10, 12, 13, 14, 15, 16, 19, 20, 23, 27, 28, 31, 32, 33, 34, 38, 39). Additionally, study ID 35 exclusively involved all female participants, while study ID 11 included only men.

### Health measures

As shown in Supplementary Table 3 (see online supplementary material), various domains, measurements, scales, and studies related to physiological health, sleep quality, perceived physical health, physical activity, and mental health were summarized. The included studies adopted various measurements and tests/scales across a range of health and wellness domains. The domain of physiological health incorporates assessments for conditions such as cardiovascular disease, hypertension, body mass index, physical function, brain-related tests, physiological stress, bone mineral density, all-cause mortality, dementia, and functional disability. Sleep quality is gauged using tools like the Pittsburgh Sleep Quality Index and Sleep Dysfunction Rating Scale. The domain of perceived physical health employs tests/scales like the Rand Medical Outcomes Study Health Survey (MOS SF-20), Visual Analogue Scale, Short-Form Health Survey (SF-36), and Short Form-12 Version 2 Health Survey (SF12v2). The mental health domain covers aspects like perceived mental health, emotions and mood state, depressive symptoms/depression, psychological distress/stress, well-being, psychological and social support, life satisfaction, and loneliness. Lastly, the quality of life is assessed using measurements such as the Nottingham Health Profile, WHOQOL-BREF, EQ-5D, and others. These domains collectively utilize an array of specific measurements and tests to holistically evaluate different facets of an individual’s health and well-being.

### Green space measures

Supplementary Table 4 (see online supplementary material) presents a detailed summary of the measures used to evaluate green and blue spaces. The objective measure of green space includes categories such as green space access, usage patterns, function of green space, coverage of green space, types/elements/variation of green space, quantity of green space, and the greening rate. These encompass measurements like the number of parks within walking distance from homes, visitation frequency, functions of the green space, coverage within specified radial buffers around participants’ homes, different types of green areas, the Normalized Difference Vegetation Index for quantity, and satellite-based urban greenery distribution for the greening rate. The subjective measure of green space includes the perception of public open space (POS), with measurements like the Perceived Restorativeness Scale, Elder-Friendly Urban Spaces Questionnaire, and perception of nine qualities of urban green space.

Similarly, the objective measure of blue space includes categories of blue space quantity, quality, access, usage pattern, function, types, and coverage. These involve measurements like the Normalized Difference Water Index, degree of fragmentation within a buffer zone, distance to the nearest water body, length of stay at the blue space, functions of coastal space, types like rivers and lakes, and coverage ratio of all types of water surfaces.

The settings for these measurements include visual stimuli like a forest landscape showing a bamboo grove, parks like Walwekar Park, and nursing centers like the Linpan study area located in Pidu District, Chengdu. These measurements help evaluate the availability, use, and benefits of green and blue spaces in urban areas.

### Relationships between green space and older adults’ health

Table 1 illustrates the relationship between different measurements of green and blue spaces and their relationships with physiological health, sleep quality, perceived physical health, and mental health. Regarding green spaces, access positively correlates with better physiological health, perceived physical health and mental health, with two studies suggesting a non-significant relationship with mental health. Green space usage patterns show a mostly positive link with all health outcomes, except for mental health, where two studies indicated a nonsignificant correlation for specific population, such as male participants. The function of green spaces is positively related to better perceived physical health and mental health. The coverage of green spaces shows mixed results; it has both significant positive and nonsignificant relationships with physiological health, perceived physical health and mental health. The type of green spaces shows a significant positive correlation with perceived physical health and mental health. The quantity of green space and the greening rate mostly show positive correlations with physiological health, perceived physical health and mental health, and quantity of green space also showed significant association with better sleep quality. Yet, the relationship between these two aspects and physiological health become nonsignificant while adjusted for control variables or among females.

**Table 1.** Health outcomes and their relation to different green space measurements.

Green space measurements	Better physiological health		Better sleep quality		Better perceived physical health		Better mental health	
	+	0	+	0	+	0	+	0
**Green space access**	1				1, 39		29	1, 29 (laopiao)
**Green space usage pattern**	1		24		1, 8, 18, 25	1	1, 18, 24, 28 (female)	1, 28 (male)
**Function of green space**					30		30	
**Coverage of green space**	4	2, 4			11	21, 31	3, 11	31
**Types of green space**					37		38	
**Quantity of green space**	16, 22, 26, 36 (male)	26 (adjusted), 36 (female)	20		14, 15		12, 13, 34	
**The greening rate**	23, 27 (male)	27 (female)			40		12, 19, 33	
**Blue space quantity**							13, 34	
**Blue space quality**							6, 7	
**Blue space access**						21		
**Blue space usage pattern**							6, 7	
**Types of blue space**					1		1	
**Coverage of blue space**						21		
**Perception of public open space**					8, 28, 32		5, 17, 28	
**Visual stimuli**							9	
**Park**							10	
**Nursing center**	35		35					

*Note*. “+” represents a significant positive relationship, “0” represents a non-significant relationship, and “–” represents a significant negative relationship.

In terms of blue spaces, quantity, quality, usage pattern, and types all show positive correlations with better mental health, whereas access and coverage relate nonsignificantly to perceived physical health. The perception of POS positively correlates with perceived physical health and mental health. Finally, visual stimuli and parks positively correlate with mental health, while nursing centers are positively correlated with physiological health and sleep quality.

### Common control variables

As shown in Supplementary Table 5 (see online supplementary material), different studies also included control variables to correct for possible bias. These control variables can be divided into five categories from individual to house, facility, and city level.

Individual demographic variables include age, sex, education, marital status, race/ethnicity, income, living area, employment status, medical cover, housing type (public or private), living arrangement (alone or with others), years of residence, hukou status, geographical region of residence, socioeconomic status, social security recipient/insurance status, assets, number of family members living together, and migration status. Individual health behavior variables encompass drinking, smoking, reported difficulty walking 100 m, functional ability, physical illness, and social and leisure activity.

House-level variables include housing facilities (presence of toilet, bathroom, water supply, and kitchen), housing construction time, per capita housing space, and distance to a major road. Facility-level variables concern the quality of care provided, facility size (number of beds), for-profit or not-for-profit status, occupancy rate, presence of a special treatment unit (e.g., Alzheimer’s), staffing per resident per day, percent female residents, percent White residents, average resident age, percent Medicaid-eligible residents as a proxy for low socioeconomic status, and assistance needs for daily living (ADL index). Finally, city-level variables include neighborhood population density, neighborhood median annual income, social deprivation index, Gross Domestic Product per square kilometer, average per capita income of the administrative area, and the proportion of older adults.

### Exposure to green space and health outcomes

As shown in Table 2, approximately half of the studies reviewed conducted a sensitivity analysis or used the distance between the living area and green/blue spaces as a moderating factor. These studies aimed to investigate whether changes in this distance would impact the relationship between green/blue spaces and health outcomes, underlining the importance of considering buffer zone size in these analyses. Among the studies that included a buffer zone analysis, most studies found that proximity to green space was associated with higher levels of perceived physical and mental health. Most of these studies reported a U-shaped relationship, with the strongest association found in the third quartile. However, there was one exception where a study found that living further from green space was not significantly associated with health outcomes (Lin & Wu, 2021).

**Table 2.** Distance from green/blue space and relationship with outcomes.

	Change of green space exposure	Studies
Green space and mental health	0–250 m, 250–500 m, 500–1,000 m, 1,000–3,000 m	3 (The strongest associations were seen for green space cover closer to facilities)
	Quartile 1–4	13 (People exposed to more green and blue space (i.e., second, third, or fourth quartile) had significantly lower GDS-15 scores than people residing in neighborhoods with a low coverage of green and blue space (i.e., first quartile). The green and blue space coefficients were most pronounced in the third quartile.)
		33 (higher exposure to green space with lower mental health issue)
	50 m, 100 m, 150 m, 200 m, 250 m	34 (inverse relationship)
	400 m, 800 m	38 (Further buffer with higher QoL)
Green space and physical health	Quartile 1–4	15 (1,000 m, 500 m, 1,500 m, 2,000 m, 3,000 m buffer; Older adults living in greener settings (quartiles 2, 3, and 4) were more likely to report good health than those living in the least green quartile areas. This association was strongest for the third quartile. The estimations found in the 1,000 m buffer were consistent with those when the NDVI was assessed using 500 m, 1,500 m, 2,000 m, and 3,000 m buffers.)
		16 (250 m, 1,250 buffer; fourth quartile least mortality)
		19 (depression increase as the ratio of the green area decreased)
		22 (300 m and 500 m buffer; u-shape)
		23 (more green space with lower risk of cardiovascular diseass (CVD)
		36 (u-shape)
	NDVI 1, 3, and 8km;	14 (more green exposure healthier)
	0–0.3, 0.3–0.5 and 0.5–1 km;	21 (more green exposure healthier)
	Quintile 1–5	4 (1,600 m and 800 m buffer; u-shape)
	<200 m, 200–400 m, 400–800 m, >800 m	31 (living further with lower level of poor outcome)
	In the living district, first-degree, second-degree, third-degree	27 (Living further with poor outcome)

### Possible mediators and moderators

**Figure 2.**
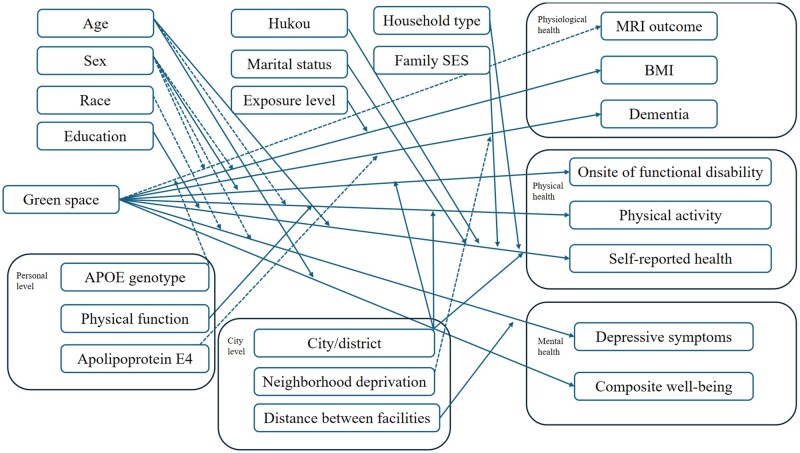
Moderation factors examined in the included studies.*Note*. Dashed lines indicate non-significant relationships, while solid lines indicate significant relationships in the studies.

**Figure 3.**
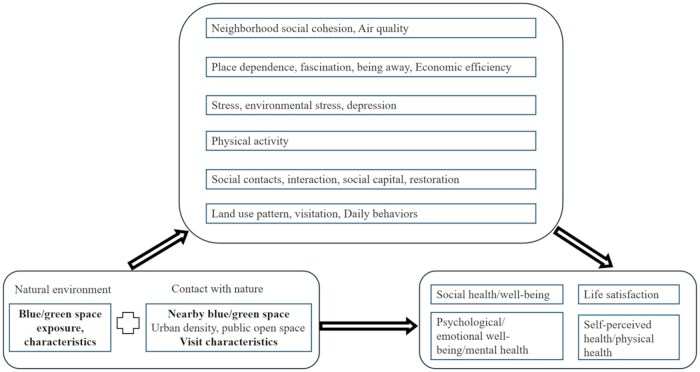
Mediation relationships examined in the included studies.

As depicted in Figures 2 and 3, the associations between green space and physiological, physical, and mental health are moderated by demographic factors (such as age, sex, education level, hukou status, marital status, household type, and family socioeconomic status), individual behavior (level of exposure), and city-level factors (such as city/district and the distance between facilities and green spaces). Concurrently, potential mediators were examined to elucidate the mechanisms behind the impact of green/blue spaces on older adults’ mental and perceived physical health. These mediators include the neighborhood environment (specifically social cohesion and natural air quality), individual perceptions of space (including feelings of dependence, fascination, escape, and economic efficiency), personal mental health status (depression, stress), physical activity levels, social capital (including social contacts, interaction, and restoration), and individual behaviors (such as land use patterns, visitation frequency, and daily behaviors).

Most of the studies did not include the variable of rural/urban differences as a moderator. Some studies were conducted in specific cities or areas (e.g., Study Nos. 4, 6, 7, 12, 13, 17, 18, 19, 28, 29, 31, 38, 39, 40), such as Guangzhou (China), Beijing (China), and Hong Kong SAR. While their findings provide insights into urban contexts, they may have limited applicability to rural settings. Other studies that did consider rural/urban differences (Study Nos. 16, 30) treated them only as control variables in descriptive analyses, without further exploration or discussion. Only two studies examined these differences in relation to green space and health among older adults: one (Study No. 11) reported no significant differences, while another (Study No. 15) found significant effects.

### Mechanisms

Our review also examined potential mediators to elucidate the mechanisms behind the impact of green and blue spaces on older adults’ mental and perceived physical health. These mediators provide valuable insights into the pathways through which natural environments may influence well-being.

Key mediators identified include aspects of the neighborhood environment, such as social cohesion and natural air quality. Social cohesion, or the sense of community and mutual support among neighbors, may enhance feelings of safety and belonging, thereby positively impacting mental health. Natural air quality, often better in areas with abundant green space, can reduce exposure to pollutants, contributing to improved physical health.

Individual perceptions of space also emerged as important mediators. Feelings of dependence, fascination, and escape, as well as perceptions of economic efficiency, reflect the subjective experience of interacting with green spaces. These perceptions can influence how individuals use and benefit from these environments, with potential impacts on mental well-being and perceived physical health. Personal mental health status, including levels of depression and stress, was another critical mediator. Green spaces may offer restorative experiences that alleviate symptoms of depression and reduce stress, thereby enhancing overall mental health. Physical activity levels were frequently cited as mediators in the relationship between green space and health. Access to green and blue spaces can encourage physical activity, which is known to improve both physical and mental health outcomes.

Social capital, including social contacts, interactions, and restorative experiences, also plays a significant role. Green spaces often serve as venues for social interaction and community engagement, which can strengthen social networks and support mental health. Finally, individual behaviors, such as land use patterns, visitation frequency, and daily behaviors, were identified as mediators. How often and in what ways individuals engage with green spaces can significantly influence the extent of health benefits derived from these environments.

These findings underscore the complexity of the relationship between green spaces and health, highlighting the importance of considering a broad range of mediating factors. Future research should continue to explore these mediators in depth to better understand the mechanisms at play. Additionally, interventions aimed at enhancing the health benefits of green spaces should consider strategies to improve social cohesion, air quality, and opportunities for physical activity, while also fostering positive individual perceptions and behaviors related to green space use.

## Discussion

To our knowledge, this is the first study to comprehensively map the evidence on the relationship between different measures of green space and diverse health outcomes among older population. Following a systematic procedure, 40 studies were eventually encompassed in this scoping review. The findings typically depicted significantly positive relationships between exposure to green/blue spaces and perceived physical and mental health. Potential confounders, moderators, and mediators were explored for understanding the mechanisms that underlie these observed relationships.

### Study design

Overall, a small portion of the studies we reviewed employed a longitudinal design, while the majority were cross-sectional. Using cross-sectional data, reverse causality cannot be ruled out, as the outcome may precede the exposure (Cohen et al., 2017). Additionally, cross-sectional studies are susceptible to self-selection bias; for example, less healthy adults might choose to move to neighborhoods with more available green space (Maas et al., 2008). Nevertheless, 4 out of the 40 studies utilized a longitudinal study design. Longitudinal studies are less prone to reverse causation and self-selection bias (Cohen et al., 2017). By providing trajectories of health status or disease incidence over time, they are better equipped to evaluate the effects of green space exposure on the aging process.

### Gender distribution of included studies

This imbalance in sex representation across studies warrants careful consideration when interpreting the effects of green space exposure on health outcomes. A predominance of female participants in many studies may influence the generalizability of findings, as men and women might respond differently to environmental exposures due to biological, psychological, and social factors (Bolte et al., 2019; Richardson & Mitchell, 2010; Sander et al., 2017; Sillman et al., 2022), and generally, women might have “more to gain” than men from living near green spaces (Sillman et al., 2022). The exclusive focus on one sex in studies ID 35 and ID 11 further highlights the need for more inclusive research designs that account for potential sex-specific differences in health responses to green space. Moreover, the underrepresentation of male participants in many studies could lead to an incomplete understanding of the broader population’s health dynamics. Future research should strive to achieve a more balanced and representative sample to enhance the robustness and applicability of findings.

### Green space measurement

By incorporating diverse metrics—function, coverage, types, quantity, and subjective perceptions, our study provided a more comprehensive understanding of green space exposure. Like the previous scoping review for general population (De Keijzer et al., 2020), which relied on objective, satellite-based green space indices, our study also revealed a substantial limitation existed in most of the studies: studies focus only on residential exposure, ignoring green spaces encountered elsewhere, which is particularly relevant for older adults who may venture beyond their neighborhoods. This reliance on broad geographical units, like postcode centroids, risks exposure misclassification. Mobility-based methods (MBM) address this by capturing individuals’ movements, offering a holistic view of green space interactions compared to traditional residence-based methods, which underestimate exposure (Kwan, 2012; Liu et al., 2023, 2024). MBM also accounts for spatial non-stationarity, where green space impacts vary across urban, suburban, or rural contexts, and temporal variations, such as daytime accessibility being more beneficial due to visibility (Liu et al., 2023). For older adults, reduced mobility and safety concerns—uneven terrain, inadequate seating, or poor lighting—limit green space access, making mobility data crucial for identifying barriers and designing accessible interventions, like walkable paths or shaded areas. Temporal patterns, such as morning green space use, further inform age-friendly designs. By leveraging mobility data, researchers can enhance exposure accuracy, optimize green spaces for health benefits, particularly for older adults, and ensure contextually relevant interventions. This approach, emphasizing spatial, temporal, and population-specific insights, will strengthen our analysis of green space–health relationships (Kwan, 2012; Liu et al., 2023, 2024).

### Relationships between green space and outcomes and pathways

The results of our review indicate that the majority of studies found significant associations between green space exposure and various health outcomes, including better physiological health, improved sleep quality, and enhanced perceived physical and mental health, which were in accordance with other studies that examined the relationships between green space and health among general population (Yang et al., 2021) and adolescents (Ye et al., 2022). These findings underscore the potential benefits of green space on overall well-being, supporting the notion that access to natural environments can play a crucial role in promoting health.

However, it is important to note that after adjusting for potential confounders, some of these significant relationships became insignificant. This suggests that while there is a general trend indicating the positive impact of green space, other factors may also influence the relationship. Our review provided valuable insights into the confounding variables considered in the included studies. These confounders spanned multiple levels, including individual-level demographic and health behavior variables, household-level variables, facility-level variables, and even city-level variables. Recently, artificial light at night (ALAN) has been identified as a significant environmental confounder in studies assessing the impact of green space on general population’s health (Liu et al., 2024). Urbanization has led to a scarcity of natural environments alongside an abundance of man-made infrastructure, intensifying the interaction and dependence between green space and artificial light at night within urban settings. In the studies focusing on older adults, the influence of ALAN on the relationship between green space and health has been overlooked, which may lead to erroneous assessments of health impacts and inadequate public health strategies.

Our study also revealed that the attenuation of associations between green space and older adults’ health after adjustment is influenced by mediators and moderators such as socioeconomic status, lifestyle behaviors (such as physical exercise and social activities), and environmental (such as air pollution, noise, and heat) factors. This finding aligns with a previous scoping review by Dzhambov et al. (2020), which also highlighted the role of these factors in the relationship between green space and health among the general population. Moreover, studies on the general population indicate that confounding variables (such as air pollution and noise pollution) reveal more complex mechanisms by influencing human behavior, daily mobility, and activities (Kan et al., 2023; Liu et al., 2023). For example, high levels of air pollution may lead individuals to reduce outdoor activities, thereby indirectly affecting physical exercise, while exposure to green space can promote social interactions and physical activity, ultimately improving health. Additionally, an individual's daily activities can influence their exposure to different environments (such as pollution and green space), leading to the complexity of health outcomes (Liu et al., 2023). Therefore, a thorough exploration of these mechanisms is essential for understanding how green space can enhance health through human behavior and environmental exposure, providing important evidence for the formulation of public health policies.

### Distance of accessing green space and health outcomes

The results presented in Table 2 reveal significant insights into the link between green/blue spaces and health outcomes, particularly focusing on the influence of the proximity of residential areas to these spaces. Interestingly, among the studies that incorporated a buffer zone analysis, a majority identified a positive correlation between proximity to green space and higher levels of perceived physical and mental health. This is consistent with the growing body of research suggesting that increased access to natural spaces can have a substantial impact on overall health and well-being. The majority of these studies reported a U-shaped relationship, with the most potent association observed in the third quartile, suggesting that there is an optimal distance from green spaces for maximizing health benefits (Lennon et al., 2017). Conversely, distances that are either too short or too large may induce too many contextual errors and fail to capture the associated health benefits (Liu et al., 2024). It underscores the complexity of this relationship and highlights the need for further research to understand the specific conditions under which proximity to green/blue spaces can have a beneficial or detrimental impact on health outcomes.

### Limitations

This scoping review examined the relationship between green space and health outcomes in older adults, but several limitations should be noted. One primary limitation is related to the assessment of study quality. Given that the focus of this review was on covering the range of work that informs the topic rather than limiting the included studies to those that meet particular standards of scientific rigor, it was not possible to determine the quality of the included studies comprehensively. As a result, the findings presented in this review may be influenced by the inclusion of studies with varying levels of methodological quality, potentially affecting the robustness of the conclusions drawn.

Additionally, while we aimed to be comprehensive in our approach, there is a possibility that not all publications relevant to the inclusion criteria were identified by the searches or databases used. The reliance on specific databases and search terms may have led to the omission of some studies, particularly those published in non-indexed journals or in languages other than English. This limitation highlights the potential for incomplete coverage of the existing literature, which could impact the generalizability and comprehensiveness of our findings.

Furthermore, the heterogeneity of the included studies in terms of their design, measures of green space, and health outcomes poses a challenge for synthesizing the results. The diverse methodologies and varying definitions of green space and health metrics across studies complicate the ability to draw consistent and overarching conclusions. This variability underscores the need for more standardized approaches in future research to facilitate better comparison and synthesis of findings.

### Future research directions

While the current scoping review has provided valuable insights into the relationships between green/blue spaces and various health outcomes in older adults, several gaps and areas for future research have been identified. One significant limitation observed is the lack of geographic context tailored specifically for older adults in the studies reviewed. Although older adults were included as participants, the included studies did not introduce measurements or activities that are specifically designed to fit the unique needs and preferences of this demographic. Future research should develop and utilize older adult-specific measures and activities that consider mobility limitations, safety concerns, and social engagement preferences to better understand how these factors influence health outcomes in older populations.

Additionally, while the studies examined the relationship between green space exposure and mental health, as well as quality of life, they largely overlooked an important dimension: the spiritual health of older adults. Given that older adults are often in the later stages of life and may be confronting end-of-life issues, spirituality can play a crucial role in their overall well-being. Spiritual health encompasses a sense of meaning, purpose, and connection, which can be profoundly influenced by natural environments. Future research should incorporate spiritual health as a specific aspect of measurement to provide a more holistic understanding of how green spaces impact the well-being of older adults. This could involve exploring how exposure to natural environments facilitates spiritual experiences, provides comfort, and enhances a sense of peace and fulfillment.

Our review revealed that few studies have explored the relationship between green space and older adults’ health outcomes, moderated by urban–rural differences, with the only two identified studies (Gong et al., 2014; Huang et al., 2022) reporting conflicting findings. Urban density, encompassing factors such as residential and building density, likely influences older adults’ interactions with green and blue spaces, potentially moderating the relationship between green space exposure and their mental and physical health. In rural settings, greater access to expansive natural environments may enhance the restorative benefits of green space, fostering physical activity and social engagement, which are critical for older adults’ well-being (Maas et al., 2009). Conversely, in urban areas, higher density may limit access to quality green spaces, potentially attenuating their health benefits, though compact green areas like pocket parks can still provide significant mental health advantages (Nordh & Østby, 2013). Exploring these rural–urban contextual differences would deepen understanding of how environmental factors shape health outcomes, informing targeted urban planning and public health interventions for aging populations.

Moreover, there is a need for more longitudinal studies to examine the long-term impacts of green and blue space exposure on the health of older adults. Such studies can help to establish causality and provide deeper insights into how continuous interaction with natural environments contributes to sustained health benefits over time. Additionally, future research should consider the use of mixed methods approaches to capture both quantitative and qualitative data, thereby offering a more comprehensive understanding of the subjective experiences of older adults in relation to green/blue spaces.

Another important dimension for future studies is the examination of people–place relations, particularly place attachment and its components (place identity and place dependence). These psychosocial factors may serve as either mediators or moderators in the green space-wellbeing relationship among older adults. For instance, stronger place attachment could (1) enhance the psychological benefits of green space through increased sense of belonging (Scannell & Gifford, 2017) or (2) motivate more frequent use of nearby nature spaces, thereby amplifying physical activity benefits (Lewicka, 2011). This perspective would provide a more holistic understanding of person-environment transactions, particularly for aging populations who may have longstanding connections to their residential environments. Future studies should incorporate validated measures of place attachment alongside green space exposure metrics to explore these potentially important mechanisms.

Most importantly, future studies should advance the “mobility turn” in green space research by transitioning from static residence-based exposure assessments to dynamic mobility-based approaches that better reflect how people interact with urban environments (Liu et al., 2023). Traditional methods frequently misrepresent true exposure since individuals’ daily movements—influenced by socio-demographic ­factors, temporal rhythms, and physical mobility constraints—fundamentally shape green space accessibility and health benefits. Emerging technologies like GPS tracking and street-view image segmentation, combined with spatiotemporal analytics, and the most updated technologies of artificial intelligence, now enable researchers to capture these complex exposure patterns more accurately (Liu et al., 2023), while revealing hidden inequalities that static measures often overlook, particularly for vulnerable groups like older adults or mobility-limited populations. Critical research priorities should focus on overcoming methodological challenges such as the neighborhood effect averaging problem through improved mobility-aware exposure metrics, while also integrating real-time movement data with health outcomes to identify optimal exposure windows and conducting place-specific studies that account for geographic variations in both green space distribution and human mobility patterns. These advances will provide the evidence base needed to develop targeted urban policies and equitable interventions that maximize green space benefits across diverse communities. By embracing these dynamic approaches, future research can generate more nuanced understandings of how people, especially older adults, actually experience and benefit from urban nature in their daily lives.

### Policy and planning implications

Our study provides value insights on how green spaces can promote healthy aging and offer several policy implications for development of interventions and urban planning strategies. First, urban planning should prioritize the development of multifunctional community green spaces tailored to older adults’ needs. These spaces should integrate exercise, social, and leisure functions, such as installing age-appropriate fitness equipment, shaded seating areas, and small social plazas. Research indicates that older adults value safety and convenience in green spaces, and multifunctional environments can simultaneously promote physical activity (e.g., walking) and social interaction (e.g., gardening clubs), which are directly linked to physical and mental health. Additionally, coupling community parks with primary healthcare facilities (e.g., community health service centers) and community centers for older people through spatial integration design can enhance both green space utilization and health management. For instance, incorporating “health stations” within green spaces that offer services like blood pressure monitoring and rehabilitation guidance can encourage more visits and improve overall well-being.

Second, there is an urgent need to establish a special policy framework for “green spaces and healthy aging.” Governments can include green space coverage and age-friendly renovation rates in the assessment metrics for healthy cities, clearly defining local authorities’ responsibilities for green space investment in aging communities. Creating cross-departmental cooperation mechanisms involving health, urban construction, transportation, and civil affairs sectors is essential. Moreover, piloting the “green prescription” model, where family doctors prescribe personalized green space activities (e.g., 30 min of park walking, three times a week) for older adults and collaborate with community senior care centers to monitor compliance, can effectively bridge the gap in spontaneous health behaviors.

Third, enhancing older adults’ awareness of the health benefits of green spaces is key to promoting their utilization. Community lectures and courses in senior universities should be organized to disseminate knowledge about “nature therapy,” presenting empirical evidence linking green space exposure to chronic disease mitigation Encouraging seniors to participate in green space design and maintenance, such as through “Silver Gardener” programs, can significantly increase their sense of ownership and willingness to use these spaces. Additionally, promoting intergenerational interaction within green spaces is essential. Designing intergenerational activity zones (e.g., adjacent children’s play areas and senior resting areas) and supporting social organizations to conduct “time bank” mutual assistance services (where younger seniors assist older ones in green space activities, accumulating service hours for future care) can strengthen social connections. Since social interaction is a critical mediator of green spaces’ mental health benefits, intergenerational integration can enhance both social support and self-efficacy among older adults.

## Conclusion

This scoping review has highlighted the significant relationship between exposure to green spaces and various health outcomes among older adults. The evidence indicates that access to green spaces contributes positively to physical, mental, and social health, with mechanisms such as increased physical activity, reduced stress, and enhanced social interaction playing key roles. However, the predominance of cross-sectional studies hampers our ability to draw causal inferences, indicating a crucial need for longitudinal research to establish stronger connections over time.

Furthermore, it is evident that current research has not adequately addressed the specific needs of older adults when examining the impacts of green spaces. Future studies should incorporate age-specific measures, consider mediators and moderators that impact the green space-health relationship, and explore the spiritual health aspects related to older adults. Overall, a more nuanced understanding of how green spaces can enhance the well-being of older adults is essential, guiding future public health and urban planning strategies that prioritize this demographic.

## Supplementary Material

igaf119_Supplementary_Data

## Data Availability

The data supporting the findings of this scoping review consist of published articles retrieved from five electronic databases: Web of Science, PubMed, Scopus, and EBSCO. The full search strategies and selection criteria are described in the Method section of the manuscript. Details of the search process, article selection, and the extracted data items are available from the corresponding author upon reasonable request.
