# Supplementary material for: Green space and older adults’ health: a scoping review
Source: Innov Aging. 2025 Oct 30;9(12):igaf119. doi: 10.1093/geroni/igaf119 (PMC12707908; doi:10.1093/geroni/igaf119)
Supplement: igaf119_Supplementary_Data [file igaf119_supplementary_data.docx]

***Innovation in Aging* Supplementary Material: Wang, Song, Liu, & Shi. Green space and older adults’ health: A scoping review.**

**Supplementary Table 1.** Search terms

| Search topics | Search |
| --- | --- |
| Green space | Green space OR green open space OR Green Public Space OR Public Open Space OR Urban Forest OR City Park OR exposure to nature |
| Health | Health OR wellbeing OR quality of life |
| Older people | Older people OR adults persons OR elderly |

**Supplementary Table 2.** Basic information of all included studies

| **ID** | **Author (year)** | **Location_ country / region** | **Study Population** | **Sample size** | **Gender** | **Mediation** | **Moderation** | **Urban/rural** |  |
| --- | --- | --- | --- | --- | --- | --- | --- | --- | --- |
| 1 | Aliyas (2019) | Iran | Older adults (65 years or above) | 978 | Men were slightly more over-represented | x | x | x |  |
| 2 | Besser et al. (2021) | US | Older adults (65 years or above) | 1125 | Women were slightly more over-represented | x | x | x |  |
| 3 | Browning et al. (2019) | US | Older adults (65 years or above) living in U.S. nursing homes | 9186 facilities | Women were slightly more over-represented | x | x | x |  |
|  |  |  |  |  |  |  |  |  |  |
| 4 | Carthy et al. (2020) | Ireland | Older adults aged over 50 | 5804 | Women were slightly more over-represented | x | x | only urban |  |
| 5 | Chen et al. (2022) | China | Young-old (aged 65 to 75) & old-old group (aged76 to 95) | 501 | more female than male | Green space and wellbeing. Mediators: NSC (neighborhood social cohesion) and PD (place dependence) | x | x |  |
|  |  |  |  |  |  |  |  |  |  |
| 6 | Chen et al. (2020) | China | Aged 60–90; | 966 |  | Hydrophilicity index and mental health mediated by stress. The association between per capita water area and mental health was mediated by pollution, stress, and social contact. | x | only urban |  |
| 7 | Chen et al. (2022) | China | Between the ages of 60 and 90 years | 966 |  | Mediators: air quality, stress, physical activity duration, and social contact. Only stress was a significant mediator in the effect of the NDWI | The relationship between neighborhood blue space and SRH varied significantly by age and income. neighborhood blue space, and SRH status. | only urban |  |
| 8 | Dahlkvist et al. (2016) | Sweden | Older people residing in care facilities | 290 | more female than male | Garden greenery and self-reported health. Mediators: being away (significant), fascination (significant), visitation (non-significant), being away + visitation (significant), being away + fascination (significant), fascination + visitation (non-significant), being away + fascination + visitation (non-significant). |  | x |  |
| 9 | Elsadek et al. (2021) | N/A | Participants aged 82.9+0.78 years | 34 | half and half | x | x | x |  |
| 10 | Gaikwad & Shinde (2018) | India | Older persons who are regular users of the park | 31 | more female than male | x | x | x |  |
| 11 | Gong et al. (2014) | UK | Older men aged 66 and over | 1010 | all male | x | Outcome: reported regular participation in physical activities; Amount of neighbourhood green space X good lower extremity physical function (vs. poor): nonsignificant; High variation in neighbourhood vegetation X good lower extremity physical function (vs. low variation and poor): significant | urban vs. non-urban: non significant |  |
| 12 | He et al. (2022) | China | Older adults aged 60 and above | 1594 | slightly more female than male | Mediators: loneliness, sense of community (only significant), frequency of physical activity, and neighborhood walk score metric. | Eye-level greenery cushioned the negative effect of urban density on life satisfaction. Floor area ratio × greening rate & Floor area ratio × NDV & Floor area ratio × accessibility to nearest parks: non-significant | only urban |  |
| 13 | Helbich et al. (2019) | China | Older adults aged 60 and above | 1190 | slightly more female than male | x | x | only urban |  |
| 14 | Huang et al. (2020) | China | Older adults aged 60 or over in China | 368399 | slightly more female than male | x | NDVI-health association was stronger in high-density urban areas relative to low-density urban areas and rural areas. Furthermore, the association was stronger for participants who were younger, higher-educated, and non-agricultural hukou holders. |  |  |
| 15 | Huang et al. (2022) | China | Older adults aged 60 or over in China | 300442 | slightly more female than male |  | The greenness-SGH association was stronger for older adults who resided in urban areas, were female, older (>= 80 years), were single or divorced or widowed, had elementary or secondary school education, and lived in households with a car valued over ¥ 200,000. Greenness can help mitigate the adverse effects of air pollution and outdoor light pollution at night on older adults’ SGH. Residential greenness can buffer against the detrimental effects of air pollution and light pollution at night on older adults’ health. However, there were no statistically significant interactions between summer mean temperature and NDVI quartiles. | Significant moderator |  |
| 16 | John et al. (2019) | China | Individuals aged 80 years or older in China, | 23754 | more female than male | x | x | included urban and rural participants but no analysis |  |
| 17 | Lak et al. (2023) | N/A | Older adults aged 60 and above | 420 | more male than female | x | x | only urban |  |
| 18 | Lau et al. (2021) | China | Older adults aged 60 and above in Hong Kong | 462 | more male than female | x | x | only urban |  |
| 19 | Lee & Lee (2019) | South Korea | Urban older population aged 65 years and older | 11408 | more female than male | x | x | only urban |  |
| 20 | Li et al. (2021) | China | Older individuals aged 60 years or above in rural areas | 2686 | more female than male | Green space was a mediating factor between CO2 and sleep quality in the mediating effect model. | The negative correlation between green space and sleep quality decreased with concentrations of CO2 increased. | x |  |
| 21 | Lin& Wu et al. (2021) | China | Older Chinese men and women aged 65 years or above | 1773 |  | x | x | x |  |
| 22 | Lin et al. (2021) | N/A | Older Chinese men and women aged 65 years and above | 3944 | almost half | x | x | x |  |
| 23 | Massa et al. (2016) | Brazil | Aged 60 or older | 1333 | more female than male | x | x | x |  |
| 24 | Rappe & Kivelä (2005) | Finland | Older individuals 70 and above | 30 |  | x | x | x |  |
| 25 | Rappe et al. (2006) | Finland | Older women living in long-term nursing care in Finland | 45 |  | x | x | x |  |
| 26 | Slawsky (2019) | US | Individuals aged 75 years or older | 3069 | more male than female | x | x | x |  |
| 27 | Takagi et al. (2022) | Japan | Older adults (aged ≥65 years) living in Japan | 363791 | more female than male | x | Moderator between the number of parks and sports facilities: gender. In particular, these associations were stronger for the adjacent district than for individuals’ living districts. | x |  |
| 28 | Tan et al. (2019) | China | 55 years and above | 326 | more female than male | x | Income was found to be a significant moderator of the association between the duration of visit and social functioning. the duration of the visit and social functioning were significantly related among old-older respondents but not their young-older counterparts. The relationship was also significant among older adults living alone but not those living with families. Furthermore, the association between the duration of UGS visits and self-rated mental health was significant in female respondents but not male respondents. | only urban |  |
| 29 | Tang et al. (2020) | China | 60 years and above | 591 | more male than female | Social capital (bonding, bridging, and linking capital) as mediator between green space and health: significant | Moderator: local older adults and laopiao | only urban |  |
| 30 | van den Berg et al. (2010) | Netherlands | Allotment gardeners and neighbors without allotments in the Netherlands |  |  | x | The impacts of allotment gardening on health and well-being were moderated by age | have urban and rural but no discussion |  |
| 31 | Vogt et al. (2015) | Germany | Participants aged 65 years or older | 1711 | more female than male | x | x | only urban |  |
| 32 | Wagner et al. (2020) | N/A | 60 years and above | 720 | more female than male | x | Moderator: city (significant) | x |  |
| 33 | Wu et al. (2015) | England | Individuals aged 74 and over | 2424 | more female than male | x | x | x |  |
| 34 | Wu & Ren (2020) | China | Individuals aged 60 years and over | 757 | more female than male | x | x | x |  |
| 35 | Ye et al. (2023) | China | 60 older women, including those with hypertension, in a rural setting | 60 | all female | x | Moderators: season (significant) and health condition (significant) | x |  |
| 36 | Yu et al. (2018) | N/A | Community-dwelling Chinese adults aged 65 years and older | 3240 | more male than female | Mediators: physical activity (significant), no. of disease (non-significant), depression (non-significant), cognitive function (non-significant) | Moderation: gender | x |  |
| 38 | Zhang et al. (2019) | N/A | living community dwellers in Hong Kong | 909 | more female than male | x | Moderator: neighbourhood environmental attributes (significant) | only urban |  |
| 39 | Zhang et al. (2021) | China | Older individuals living in high-density built environments | 882 | more female than male | Physical activity and social networks play a separate role in mediating the effect of the built environment on older individuals’ physical health. | x | only urban |  |
| 40 | Zhang et al. (2014) | China | living adults aged 60 and over | 274 |  | x | x | only urban |  |

**Supplementary Table 3.** Health outcomes adopted by included studies.

| **Domain** | **Measurements** | **Tests/Scales** | **Studies** |
| --- | --- | --- | --- |
| Physiological health | Cardiovascular disease | Confirmed by a medical diagnosis | 1, 23 |
|  |  | HRV measured by the wireless multisensory  device | 9 |
|  |  | 5-item Cardiovascular Health Study (CHS) frailty phenotype | 36 |
|  | Blood pressure and hypertension | Tested by trained students after 5 minutes of participants’ rest or by devices | 1, 35 |
|  | Finger blood oxygen |  | 35 |
|  | Saliva samples |  | 35 |
|  | Body Mass Index (BMI) | Data obtained from health assessment | 4 |
|  | Physical function | the difficulty of lower extremity strength and balance | 11 |
|  | Brain-related test | MRI scan for white matter grade and ventricle grade | 2 |
|  |  | EEG test by using the Emotive EPOC wireless EEG headset | 9 |
|  | Physiological stress | Skin conductance measured by using a portable skin electrodermal sensor | 9 |
|  | Bone mineral density | Measured by the dual-energy X-ray absorptiometry | 22 |
|  | All-cause mortality | Reported by the next of kin | 16 |
|  | Dementia/AD/VaD | Diagnosed by professionals | 26 |
|  | Functional disability | Physicians or staff perform standardized assessment | 24, 27 |
| Sleep quality |  | Single item question | 24 |
|  |  | Pittsburgh Sleep Quality Index (PSQI) | 20 |
|  |  | shorter sleep dysfunction rating scale (SDRS) | 35 |
| Perceived physical health | Self-reported health /Perceived physical health | Rand Medical Outcomes Study Health Survey (MOS SF-20) (Rand MOS SF-20, 4 items related to physical health) | 1 |
|  |  | The visual analogue scale (VAS) developed by the EuroQual (EQ) group (EQ-VAS) | 8 |
|  |  | 36-item Short-Form Health Survey (SF-36) | 7, 30, 39 |
|  |  | Short Form-12 Version 2 Health Survey (SF12v2) | 18, 28 |
|  |  | Single or multiple item self-reported health | 11, 14, 15, 21, 25, 30, 31, 37 |
|  |  | Self-designed standardized and structured questionnaire (BMI, history of chronic illness, etc.) | 22 |
|  | Minor health problems | Common minor health problems | 30, 33 |
|  |  | German version of the Health Assessment Questionnaire Disability Index | 31 |
|  | Physical activity | Walking, jogging, exercise, yoga, etc. | 1, 10, 11, 30, 40 |
|  |  | Park-based physical activity | 32 |
| Mental health | Perceived mental health | Rand Medical Outcomes Study Health Survey (MOS SF-20) (Rand MOS SF-20, 6 items related to mental health) | 1, 18 |
|  |  | Single item scale of self-reported mental health | 29 |
|  | Emotions and mood state | Semantic differential (SD) questionnaire | 9 |
|  |  | Profile of Mood States (POMS). | 9 |
|  | Depressive symptoms/Depression | PHQ-9 | 3 |
|  |  | Geriatric Depression Scale (GDS-15) | 13, 34 |
|  |  | Single item question | 19 |
|  |  | The Zung self-rating depression scale (ZSDS) | 24 |
|  |  | GMS | 33 |
|  | Psychological distress/stress | Single or multiple items question of subjective stress | 19, 30 |
|  |  | General Health Questionnaire (GHQ-30) | 11 |
|  | Well-being | The well-being scale (emotional, social and psychological well-being) | 5 |
|  |  | 36-item Short-Form Health Survey (SF-36, five items related to mental health) | 6 |
|  | Psychological and social support | Social contacts with friends, family, etc | 10, 30, 32 |
|  |  | Self-Rated Social Health of Iranians Questionnaire | 17 |
|  | Life satisfaction | Satisfaction with Life Scale (SWLS) | 12 |
|  |  | Life Satisfaction Index (LSI) | 30 |
|  | Loneliness | Loneliness index | 30 |
|  | Quality of life | Nottingham Health Profile | 25 |
|  |  | Simplified Iranian version of Medical Outcomes Study Short Form 12 (SF-12 v2) Health Survey | 17 |
|  |  | WHOQOL-BREF | 38 |
|  |  | EQ-5D | 31 |

**Supplementary Table 4.** Categorization of green and blue space in included studies

| **Domain** | **Category** | **Measurement** | **Studies** |
| --- | --- | --- | --- |
| Objective measure of green space | Green space access | How many parks are located within a 5/10-minute walk from their homes | 1, 10 |
|  |  | Barriers accessing green space | 8 |
|  |  | The distance from the centroid of residential neighborhoods to the nearest park | 12, 21, 26, 29, 34, 39 |
|  | Usage pattern | Green space visitation frequency: frequency of visiting the park, outdoors | 1, 8, 10, 18, 24, 25, 28 |
|  |  | Length of stay at the green space: how long they stayed at the park | 1, 18, 28, 30 |
|  |  | Time of visiting the green space | 28 |
|  | Function of green space | Green space that provides space for sitting, playing, and socializing, transport | 1, 10, 18, 30 |
|  | Coverage of green space | 1-km and 5-km radial buffers (all land uses corresponding to vegetation) around participants’ homes | 2, 21 |
|  |  | The average percentage of tree canopy cover in 30m pixels within concentric circles around each facility | 3 |
|  |  | Footpath-accessible network buffers” covering 1600m and 800m spaces and “footpath-accessible street-side buffers” of the same sizes. | 4 |
|  |  | In Euclidean or straight-line distances buffer within 500 m of the centroid of a neighborhood using ArcGIS | 17 |
|  |  | All public green space, parks, and (landscaped) cemeteries larger than 0.5 hectare within the municipal area. | 31 |
|  | Types/elements/ variation of green space | Green areas, forest areas, water areas; trees, shrubs, lawn, flowers, etc. | 8, 11, 13, 30, 37, 38 |
|  | Quantity of green space/greening rate | Normalized Difference Vegetation Index (NDVI) | 11, 12, 13, 14, 15, 16, 20, 22, 26, 34, 36 |
|  |  | The satellite-based urban greenery distribution | 12 |
|  |  | The average level of greenery from satellite images and street-view images (SVIs) within the neighborhood. | 12 |
|  |  | Ratio of urban green area per administrative area | 19, 40 |
|  |  | Total green area per square meter | 23 |
|  |  | Number of parks and sports facilities per 1000 older adults in each school district | 27 |
|  |  | The percentage of green space and private gardens in each lower-layer super output areas | 33 |
|  |  | PSF: proportion of pervious area per neighborhood | 34 |
| Objective measure of blue space | Blue space quantity | Normalized Difference Water Index (NDWI), Modified Normalized Difference Water Index | 6, 7, 13, 34 |
|  | Blue space quality | The degree of fragmentation of blue spaces within the 1 km buffer zone of the neighborhood boundary | 6, 7 |
|  | Blue space access | The distance to the nearest water body is the Euclidean distance between the centroid of residential neighborhood and the nearest river | 6, 7 |
|  |  | The closest linear distance to different types of blue spaces | 21 |
|  | Usage pattern | Length of stay at the blue space | 6, 7 |
|  |  | Activities at blue space (The hydrophilicity index) | 6, 7 |
|  |  | Function of blue space: coastal space that provides space for sitting, playing, and socializing | 1 |
|  | Types of blue space | Rivers, lakes, etc. | 13 |
|  | Coverage of blue space | Coverage ratio of all types of water surfaces with a 1-km buffer from the centroid of respondents’ neighborhoods. | 21 |
| Subjective measure of green space | Perception of public open space (POS) | Five items measuring perceptions of POS | 5 |
|  |  | The Perceived Restorativeness Scale: residents experienced being away and fascination while visiting the outdoor space | 8 |
|  |  | Elder-Friendly Urban Spaces Questionnaire (EFUSQ) | 17 |
|  |  | Perception of nine qualities of urban green space | 18 |
|  |  | Self-rated importance of elements of the garden | 24 |
|  |  | Environmental satisfaction (quality and characteristics of UGSs) | 28 |
|  |  | Perceived features of the green space (safety, attractiveness, perceived distance, etc.) | 28, 32 |
| Settings | Visual stimuli | A forest landscape showing bamboo grove | 9 |
|  | Park | Walwekar Park | 10 |
|  |  | District parks, street parks, and green spaces in public housing estates | 18 |
|  | Nursing center | The Linpan study area located in Pidu District, Chengdu | 35 |

**Supplementary Table 5.** Control variables in the included studies

| **Categories** | **Control variables** |
| --- | --- |
| Individual demographic | Age, sex, education, and marital status, race/ethnicity, income, living area, employment status, medical cover, housing type (i.e., public or private), living arrangement (alone or with others), and years of residence, hukou status, geographical region of residence, socioeconomic status, social security recipient/insurance, assets, number of family members living together, migration |
| Individual health behavior | Drinking, smoking, reported difficulty walking 100m, functional ability, physical illness, social and leisure activity |
| House level variables | Housing facilities (the presence of toilet, bathroom, water supply and kitchen), housing construction time, per capita housing space, distance to a major road |
| Facility level variables | Quality of providing care, facility size (number of beds), for-profit or not-for-profit status, occupancy rate, presence of a special treatment unit (e.g., Alzheimer’s), staffing per resident per day, percent female residents, percent White residents, average resident age, percent Medicaid-eligible residents as a proxy for low socioeconomic status, and assistance needs for daily living (ADL index), |
| City level variables | Neighborhood population density, neighborhood median annual income, social deprivation index, Gross Domestic Product (GDP) per square kilometer, average per capita income of the administrative area, proportion of older adults |

**Reference list of all included articles ( *n* = 40):**

Aliyas, Z. (2021). Physical, mental, and physiological health benefits of green and blue outdoor spaces among elderly people. *International journal of environmental health research*, *31*(6), 703-714. <https://doi.org/10.1080/09603123.2019.1681379>

Besser, L. M., Lovasi, G. S., Michael, Y. L., Garg, P., Hirsch, J. A., Siscovick, D., ... & Longstreth, W. T. (2021). Associations between neighborhood greenspace and brain imaging measures in non-demented older adults: the Cardiovascular Health Study. *Social psychiatry and psychiatric epidemiology*, *56*, 1575-1585. <https://doi.org/10.1007/s00127-020-02000-w>

Browning, M. H., Lee, K., & Wolf, K. L. (2019). Tree cover shows an inverse relationship with depressive symptoms in elderly residents living in US nursing homes. *Urban forestry & urban greening*, *41*, 23-32. <https://doi.org/10.1016/j.ufug.2019.03.002>

Carthy, P., Lyons, S., & Nolan, A. (2020). Characterising urban green space density and footpath-accessibility in models of BMI. *BMC Public Health*, *20*, 1-12. <https://doi.org/10.1186/s12889-020-08853-9>

Chen, S., Sun, Y., & Seo, B. K. (2022). The effects of public open space on older People’s well-being: from neighborhood social cohesion to place dependence. *International journal of environmental research and public health*, *19*(23), 16170. <https://doi.org/10.3390/ijerph192316170>

Chen, Y., & Yuan, Y. (2020). The neighborhood effect of exposure to blue space on elderly individuals’ mental health: A case study in Guangzhou, China. *Health & Place*, *63*, 102348. <https://doi.org/10.1016/j.healthplace.2020.102348>

Chen, Y., Yuan, Y., & Zhou, Y. (2022). Exploring the association between neighborhood blue space and self-rated health among elderly adults: Evidence from guangzhou, China. *International Journal of Environmental Research and Public Health*, *19*(23), 16342. <https://doi.org/10.3390/ijerph192316342>

Dahlkvist, E., Hartig, T., Nilsson, A., Högberg, H., Skovdahl, K., & Engström, M. (2016). Garden greenery and the health of older people in residential care facilities: A multi‐level cross‐sectional study. *Journal of advanced nursing*, *72*(9), 2065-2076. <https://doi.org/10.1111/jan.12968>

Elsadek, M., Shao, Y., & Liu, B. (2021). Benefits of indirect contact with nature on the physiopsychological well-being of elderly people. *HERD: Health Environments Research & Design Journal*, *14*(4), 227-241. <https://doi.org/10.1177/19375867211006654>

Gaikwad, A., & Shinde, K. (2019). Use of parks by older persons and perceived health benefits: A developing country context. *Cities*, *84*, 134-142. <https://doi.org/10.1016/j.cities.2018.08.001>

Gong, Y., Gallacher, J., Palmer, S., & Fone, D. (2014). Neighbourhood green space, physical function and participation in physical activities among elderly men: the Caerphilly Prospective study. *International Journal of Behavioral Nutrition and Physical Activity*, *11*, 1-11. <https://doi.org/10.1186/1479-5868-11-40>

He, D., Miao, J., Lu, Y., Song, Y., Chen, L., & Liu, Y. (2022). Urban greenery mitigates the negative effect of urban density on older adults' life satisfaction: Evidence from Shanghai, China. *Cities*, *124*, 103607. <https://doi.org/10.1016/j.cities.2022.103607>

Helbich, M., Yao, Y., Liu, Y., Zhang, J., Liu, P., & Wang, R. (2019). Using deep learning to examine street view green and blue spaces and their associations with geriatric depression in Beijing, China. *Environment international*, *126*, 107-117. <https://doi.org/10.1016/j.envint.2019.02.013>

Huang, B., Huang, C., Feng, Z., Pearce, J. R., Zhao, H., Pan, Z., & Liu, Y. (2021). Association between residential greenness and general health among older adults in rural and urban areas in China. *Urban Forestry & Urban Greening*, *59*, 126907. <https://doi.org/10.1016/j.ufug.2020.126907>

Huang, B., Yao, Z., Pearce, J. R., Feng, Z., Browne, A. J., Pan, Z., & Liu, Y. (2022). Non-linear association between residential greenness and general health among old adults in China. *Landscape and Urban Planning*, *223*, 104406. <https://doi.org/10.1016/j.landurbplan.2022.104406>

Ji, J. S., Zhu, A., Bai, C., Wu, C. D., Yan, L., Tang, S., ... & James, P. (2019). Residential greenness and mortality in oldest-old women and men in China: a longitudinal cohort study. *The Lancet Planetary Health*, *3*(1), e17-e25. <https://doi.org/10.1016/s2542-5196(18)30264-x>

Lak, A., Khodakarim, S., Myint, P. K., & Baradaran, H. R. (2023). The influencing factors of elder-friendly public open spaces promoting older adults’ health in deprived urban neighborhoods: Partial Least Square Structural Equation Modeling approach. *Frontiers in public health*, *11*, 1143289. <https://doi.org/10.3389/fpubh.2023.1143289>

Lau, K. K. L., Yung, C. C. Y., & Tan, Z. (2021). Usage and perception of urban green space of older adults in the high-density city of Hong Kong. *Urban Forestry & Urban Greening*, *64*, 127251. <https://doi.org/10.1016/j.ufug.2021.127251>

Lee, H. J., & Lee, D. K. (2019). Do sociodemographic factors and urban green space affect mental health outcomes among the urban elderly population?. *International journal of environmental research and public health*, *16*(5), 789. <https://doi.org/10.3390/ijerph16050789>

Li, G., Zhu, Z., Hu, M., He, J., Yang, W., Zhu, J., ... & Huang, F. (2022). Effects of carbon dioxide and green space on sleep quality of the elderly in rural areas of Anhui Province, China. *Environmental Science and Pollution Research*, 1-12. <https://doi.org/10.1007/s11356-021-17296-7>

Lin, C., & Wu, L. (2021). Green and blue space availability and self-rated health among seniors in China: evidence from a national survey. *International journal of environmental research and public health*, *18*(2), 545. <https://doi.org/10.3390/ijerph18020545>

Lin, J., Leung, J., Yu, B., Woo, J., Kwok, T., & Lau, K. K. L. (2021). Association of green space with bone mineral density change and incident fracture in elderly Hong Kong Chinese: Mr. OS and Ms. OS study. *Environmental research*, *201*, 111547. <https://doi.org/10.1016/j.envres.2021.111547>

Massa, K. H. C., Pabayo, R., Lebrão, M. L., & Chiavegatto Filho, A. D. P. (2016). Environmental factors and cardiovascular diseases: the association of income inequality and green spaces in elderly residents of São Paulo, Brazil. *BMJ open*, *6*(9), e011850. <https://doi.org/10.1136/bmjopen-2016-011850>

Meng, L., Wen, K. H., Zeng, Z., Brewin, R., Fan, X., & Wu, Q. (2020). The impact of street space perception factors on elderly health in high-density cities in Macau—Analysis based on street view images and deep learning technology. *Sustainability*, *12*(5), 1799. <https://doi.org/10.3390/su12051799>

Rappe, E., & Kivelä, S. L. (2005). Effects of garden visits on long-term care residents as related to depression. *HortTechnology*, *15*(2), 298-303. <https://doi.org/10.21273/horttech.15.2.0298>

Rappe, E., Kivelä, S. L., & Hannu, R. (2006). Visiting outdoor green environments positively impacts self-rated health among older people in long-term care. *HortTechnology.*, *16*(1), 55. <https://doi.org/10.21273/horttech.16.1.0055>

Slawsky, E., Fitzpatrick, A., Rhew, I., Hajat, A., Leary, C., Russette, H., ... & Kaufman, J. (2019). An Evaluation of Greenspace Exposure as a Protective Factor in Dementia Progression among US Adults 75 Years or Older. *Environmental Epidemiology*, *3*, 373-374. .<https://doi.org/10.1097/01.ee9.0000610152.42888.18>

Takagi, D., Kondo, N., Tsuji, T., & Kondo, K. (2022). Parks/sports facilities in local communities and the onset of functional disability among older adults in Japan: The J-shaped spatial spillover effects. *Health & Place*, *75*, 102801. <https://doi.org/10.1016/j.healthplace.2022.102801>

Tan, Z., Lau, K. K. L., Roberts, A. C., Chao, S. T. Y., & Ng, E. (2019). Designing urban green spaces for older adults in Asian cities. *International journal of environmental research and public health*, *16*(22), 4423. <https://doi.org/10.3390/ijerph16224423>

Tang, S., Lee, H. F., & Feng, J. (2022). Social capital, built environment and mental health: a comparison between the local elderly people and the ‘laopiao’in urban China. *Ageing & Society*, *42*(1), 179-203. <https://doi.org/10.1017/s0144686x2000077x>

Van Den Berg, A. E., van Winsum-Westra, M., De Vries, S., & Van Dillen, S. M. (2010). Allotment gardening and health: a comparative survey among allotment gardeners and their neighbors without an allotment. *Environmental Health*, *9*, 1-12. <https://doi.org/10.1186/1476-069x-9-74>

Vogt, S., Mielck, A., Berger, U., Grill, E., Peters, A., Döring, A., ... & Maier, W. (2015). Neighborhood and healthy aging in a German city: Distances to green space and senior service centers and their associations with physical constitution, disability, and health-related quality of life. *European journal of ageing*, *12*, 273-283. <https://doi.org/10.1007/s10433-015-0345-0>

Wagner, P., Duan, Y. P., Zhang, R., Wulff, H., & Brehm, W. (2020). Association of psychosocial and perceived environmental factors with park-based physical activity among elderly in two cities in China and Germany. *BMC public health*, *20*, 1-11. <https://doi.org/10.1186/s12889-019-8140-z>

Wu, Y. T., Prina, A. M., Jones, A., Matthews, F. E., Brayne, C., & Cfas, M. R. C. (2015). Older people, the natural environment and common mental disorders: cross-sectional results from the Cognitive Function and Ageing Study. *BMJ open*, *5*(9), e007936. <https://doi.org/10.1136/bmjopen-2015-007936>

Ye, X., Dou, Z., Jiang, M., Luo, Z., Li, M., Tang, H., ... & Feng, Y. (2023). Effects of Linpan nature therapy on health benefits in older women with and without hypertension. *Frontiers in Public Health*, *11*, 1208481. <https://doi.org/10.3389/fpubh.2023.1208481>

Yu, R., Wang, D., Leung, J., Lau, K., Kwok, T., & Woo, J. (2018). Is neighborhood green space associated with less frailty? Evidence from the Mr. and Ms. Os (Hong Kong) study. *Journal of the American Medical Directors Association*, *19*(6), 528-534. <https://doi.org/10.1016/j.jamda.2017.12.015>

Zhang, C. J., Barnett, A., Johnston, J. M., Lai, P. C., Lee, R. S., Sit, C. H., & Cerin, E. (2019). Objectively-measured neighbourhood attributes as correlates and moderators of quality of life in older adults with different living arrangements: the ALECS cross-sectional study. *International Journal of Environmental Research and Public Health*, *16*(5), 876. <https://doi.org/10.3390/ijerph16050876>

Zhang, R., Liu, S., Li, M., He, X., & Zhou, C. (2021). The effect of high-density built environments on elderly individuals’ physical health: A cross-sectional study in Guangzhou, China. *International Journal of Environmental Research and Public Health*, *18*(19), 10250. <https://doi.org/10.3390/ijerph181910250>

Zhang, Y., Li, Y., Liu, Q., & Li, C. (2014). The built environment and walking activity of the elderly: an empirical analysis in the Zhongshan metropolitan area, China. *Sustainability*, *6*(2), 1076-1092. <https://doi.org/10.3390/su6021076>

Zhifeng, W., & Yin, R. (2021). The influence of greenspace characteristics and building configuration on depression in the elderly. *Building and Environment*, *188*, 107477. <https://doi.org/10.1016/j.buildenv.2020.107477>
